# Current status of *Schistosoma mansoni* and the factors associated with infection two years following mass drug administration programme among primary school children in Mwea irrigation scheme: A cross-sectional study

**DOI:** 10.1186/s12889-015-1991-z

**Published:** 2015-08-01

**Authors:** Janet Masaku, Nancy Madigu, Collins Okoyo, Sammy M. Njenga

**Affiliations:** Esatern and Southern Africa Centre of International Parasite Control (ESACIPAC), Kenya Medical Research Institute (KEMRI), P.O Box 54840 - 00200, Nairobi, Kenya; Great Lakes University of Kisumu (GLUK), P.O. Box 60827, 00200 Nairobi, Kenya

**Keywords:** Schistostomiasis, *S. mansoni*, Prevalence, Re-infection, Intervention

## Abstract

**Background:**

Schistosomiasis is a major public health problem in Kenya as well as in many other tropical countries and is considered one of the most prevalent diseases in the rural population. Between 2004 and 2009, primary school children in Mwea irrigation scheme were treated for *Schistosoma mansoni*. In the four year control programme, there was occurrence of light re-infection with *S. mansoni*. Therefore, the aim of this study was to assess the current prevalence of *S. mansoni*, infection two years after the withdrawal of mass drug administration (MDA) programme.

**Methods:**

We carried out a cross-sectional study on a population of 387 children attending 3 primary schools located in Mwea irrigation scheme. Children, aged 8–16 years were interviewed and screened for *S. mansoni* using duplicate Kato-Katz thick smears. Comparisons of prevalence by age group and gender were tested for significance on the basis of the Wald test. Best prediction factors for infection with *S. mansoni* were selected using forward – stepwise variable selection method.

**Results:**

The overall prevalence of *S. mansoni* was 53.7 %, (95%CI: 49.0–59.0, p-value = 0.000). Male children had higher prevalence of infection, 66.1 % (95%CI: 59.8–73.2, p-value = 0.000) compared to females. The gender (sex) of a child was the only factor reported to be significantly associated with *S. mansoni* infection, (OR = 1.9, p-value = 0.015, 95%CI: 1.13–3.21).

**Conclusions:**

There was high prevalence of *S. mansoni* infections in the study area, two years after the withdrawal of MDA programme. We suggest that treatment should be continued in the school children at regular intervals, monitoring and surveillance intensified to ensure interruption of transmission areas.

## Background

Human schistosomiasis, is a disease caused by chronic infection with parasitic trematodes of the genus *Schistosoma*. It is endemic in 78 tropical and subtropical countries [1]. Globally, an estimated 779 million people are at risk of schistosomiasis, more than 230 million are infected, 120 million are symptomatic and 20 million suffer from severe and debilitating forms of schistosomiasis [2–5]. The burden of the disease is essentially concentrated in Africa, with an estimated 200 million people infected where more than 90 % of the infections occur worldwide [5–7]. Schistosomiasis is intimately connected with poverty, and hence, the disease delays the social and economic development in endemic countries [5, 8–10]. In Kenya, more than six million people, or approximately 23 % of the total population, are infected with urinary or intestinal schistosomiasis [2].

Presently, schistosomiasis control strategies focus on mass drug administration of praziquantel (PZQ), with special emphasis on treating school age children [11]. Schistosomiasis has adverse effects on the overall health, school attendance and academic performance of school age children particularly in developing countries [12]. Effective drugs for the elimination of these worms are available, but individuals are readily re-infected when exposed again after treatment. Various intervention schemes have been attempted to control infections in heavily exposed populations, but they have been met with limited success in under-developed areas because of the constant re-exposure to infection [2].

Encouragingly, over the past decade, efforts to control schistosomiasis which is one of the neglected tropical diseases (NTDs) have been scaled up [13]. In early 2012, the World Health Organization (WHO) issued a goal to control schistosomiasis globally by the year 2020 and put forward a roadmap as to how this could be achieved [14]. A number of influential public and private organizations now support this goal and contributed to the London Declaration [15]. In May 2012, the World Health Assembly (WHA) resolution 65.21 was adopted, which encourages member states and the international community not only to make available the necessary and sufficient means and resources in terms of medicines, but also in terms of water, sanitation, and hygiene interventions [16]. While preventive chemotherapy is considered as the mainstay of schistosomiasis control [17–19], there is considerable evidence that control packages by integrating chemotherapy, provision of clean water and improved sanitation, snail control and behavior change, when readily adapted to local settings are necessary for sustainable control and elimination of schistosomiasis [6, 20–22]. Support from national governments, institutions and the local population coupled with inter-sectoral collaboration between the health, education sectors, water and sanitation are key features to achieve sustainable control of schistosomiasis [8, 23–25].

In Kenya, schistosomiasis occurs mostly in western, coast, and selected foci in central part of the country. In central and western Kenya, schistosomiasis is predominantly caused by *Schistosoma mansoni*. Previous studies indicate that there is a direct relationship between the prevalence of *S. mansoni* and distance to Lake Victoria, such that schools within 5 km from the lakeshore can confidently be provided with mass treatment [26]. While, on the coastal region of Kenya, schistosomiasis is exclusively caused by *Schistosoma haematobium* [27, 28].

In 2005, Kenyan Ministries of Health and Education initiated a parasite control programme with the aid of Japan International Co-operation Agency (JICA) and Kenya Medical Research Institute (KEMRI). The programme was targeting *S. mansoni* and Soil transmitted helminthes (STHs) in school age children. After sensitizing and educating the community health officers and education officers in the district, 43,928 school age children from 86 schools were de-wormed with praziquantel and albendazole by trained school teachers [29]. Prior to the de-worming, baseline prevalence and intensity of parasitic infections were determined through examination of stool samples of class three children (age range 9–14 years). A follow up study of 5 cohort primary schools was carried out to monitor the effectiveness of the control programme for four consecutive years. The prevalence of the parasitic infections in the five cohort schools was 38 % for *S. mansoni* before treatment [30]. However, there was an overall parasitic re-infection rate of 16 % for *S. mansoni,* six months after treatment. The trend of re-infectioncontinued after treatment to 22 % in the second year, 31 % in the third year and 17 % in the fourth year [30]. The purpose of this study was to determine the prevalence of *S. mansoni* infection and the associated risk factors two years after withdrawal of four years of mass drug administration (MDA) programme among primary school children in Mwea.

## Methods

### Study area

**Fig 1:**
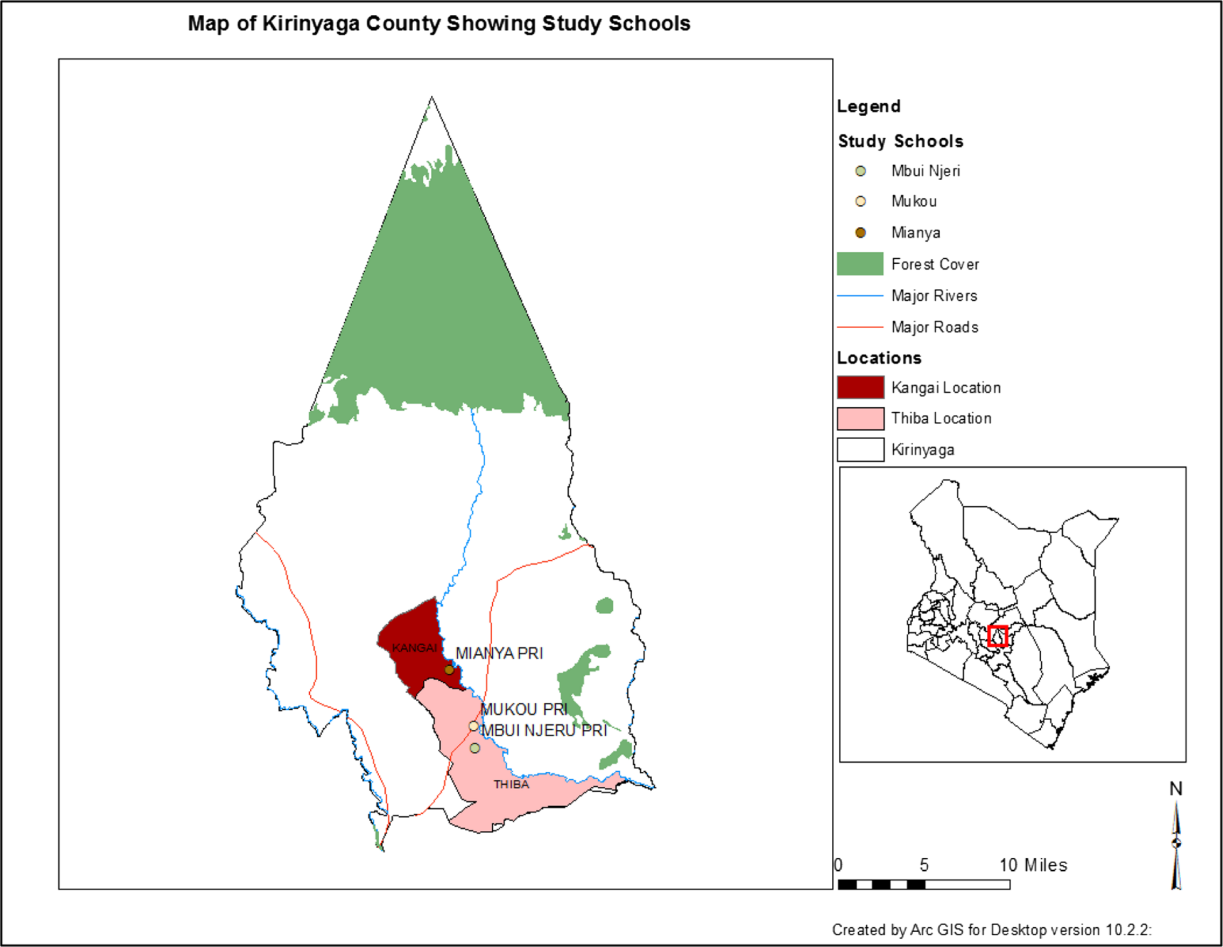
The geographical locations of the study schools

The study was conducted in Mwea irrigation scheme located in Kirinyaga County, central Kenya. Administratively, the new upgraded Kirinyaga County has two districts (Mwea East and Mwea West). The county is located about 100 km north east of Nairobi, Kenya. It covers an area of 513 km^2^ and it is estimated to have 51,444 households and a total population of 176,261 persons [31]. There are 58,970 school age children (5–19) in Mwea [31]. The mean annual rainfall in this area is in the range of 1200–1600 mm per year and varies by the time of year (Fig. 1). Mwea West district, were the study was conducted has two locations (Kangai and Thiba) and seven villages. The main socio-economic activity in this area is rice farming, which is done by gravity flow irrigation using water from river Thiba and Nyamindi. Mwea west district is endemic for both *S. mansoni* and Soil transmitted helminths (STH). The geographical locations of thesurveyed schools and the two locations are shown in Fig. 1 below.

### Study population

The target population was 390 children aged 8–16 years in three primary schools namely Mianya, Mukou and Mbui njeru. The district was purposively sampled based on schistosomiasis endemicity owing to previous studies conducted in the area [30]. All the participating pupils were to be interviewed and provide stool samples.

### Study design

This cross-sectional study was conducted in January 2012. The inclusion criteria of the schools were to be a full grade primary school, to be located within the irrigation scheme and to have participated in the MDA programme and follow up studies. Each school was treated as a cluster. In each cluster, 5 schools participated in the cohort studies and only 3 met the inclusion criteria. Probability proportional sampling for each class was used to calculate the number of children from each school to participate in the study. A total number of 642 children in class 4–7 met the inclusion criteria and were targeted to participate in the study. The sample size for the study was calculated to be 386 using the formula by Fisher et al. [32]. Study subjects were selected using simple random sampling method. In order to account for attrition or inability to produce stool specimen when required, the sample size was adjusted by 1 % to obtain a minimum sample of size 390. In the three schools, gender ratio was considered by recruiting equal number of boys and girls from each participating classes with 195 girls and 195 boys. Parent/guardian and teachers association meetings were held in all selected schools prior to the survey for communicating the study purpose and obtaining their consent. A written informed consent for participation in the study was obtained from the participants’ parents or guardians before conducting the study. In addition, assent was sought (verbal) from all the participating children before conducting the interviews and collecting stool samples.

### Sample collection

All the children with a written informed consent from their parents or legal guardians were provided with poly pots (stool containers) a day before the survey and requested to give their own fresh stool sample on collection day. The school health teacher or class teacher guided students on stool sample collection during container distribution. A trained field worker visited the school during morning break time with a registration sheet to ensure all students provided stool samples. After collection, stool samples were given unique numbers. The name, sex, age and class of each child were recorded. The specimens collected were transported to Kimbimbi Sub-District hospital for analysis. Screening of schistosome’s ova was based on duplicate Kato-Katz thick smears of 41.7 mg prepared from fresh stool samples to determine the prevalence of *S.mansoni* [33]. This procedure was carried out by trained medical laboratory technologists from the Ministry of Health (MOH).

### Questionnaire survey/household information survey

A structured pretested questionnaire developed in English and translated into Kiswahili language was administered to all the school children recruited in the study. The questionnaire was made up of demographic section including name, age, sex and name of care giver/guardian, socio-economic indicators such as level of education of their parent, occupation and property owned by their parent. The relative socio-economic status of a household was determined by possession of the following indicators, radio, television and electricity. Any study participant from a household owning one or more of these was considered as being in the relatively high category. Environmental indicators like, whether the school children had a pit latrine at home, type of housing at home (wall and floor), source of water for drinking in their homestead and preferred way or place of defecation while participation in rice farming. The last section was on health indicators, which included, receiving deworming drugs (where, when and how many times). Also pupils were interviewed on school absenteeism due to illness, action taken, distance to the nearest health facility (time taken by walking) and cost of treatment.

### Ethical considerations

The study was reviewed and approved by the Scientific Steering Committee and Ethical Review Committees of the Kenya Medical Research Institute (KEMRI). Children infected with schistosomes were treated with 40 mg/kg praziquantel by a clinical officer in accordance with WHO guidelines [34].

### Data management and analysis

Data collected was counter-checked for accuracy and verified before double entry into a computer Excel spreadsheet. All statistical analyses were survey set and carried out using STATA version 12.0 (STATA Corporation, College Station, TX, USA) and the map was developed using ArcGIS for desktop version 10.2.2 (Esri Inc. USA). The observed overall prevalence of *S. mansoni* was calculated, by school, location, gender, class and age group levels. Confidence interval of 95 % (95%CI) was obtained by binomial logistic regression, taking into account clustering by schools. Comparisons of prevalence by age group and gender were tested for significance on the basis of the Wald test. For purposes of this analysis, the following age groups were used: 8–9, 10–11, 12–13 and >13 year olds.

The significance of the factors associated with *S. mansoni* infection in the school children was determined using bivariable and multivariable logistic regression model reporting the odds ratio at 95%CI. The choice of the model was based on the *Akaike Information Criterion (AIC)* method which was used to compare between linear regression and logistic regression models. The latter was picked since it reported the least AIC value. These two models were all based on *log-likelihood function*, given the two models, the one with smaller AIC fits the data better than the one with larger AIC. Best prediction factors for the infection were subsequently selected using forward – stepwise variable selection method.

## Results

The overall data was collected from 387 children in 3 primary schools in 2 locations of Mwea West district. However, 3 children did not provide stool samples and were subsequently removed from the analysis. Majority of children surveyed 156 (40.31 %) and 155 (40.05 %) were between age groups (12–13) and (10–11) years respectively. These children were equally distributed according to class/standard. The mean age of pupils was 12 years (SD = 1.5 years) with age range of 8–16 years. The study participants had slightly equal representation in terms of gender, 192 (49.61 %) males and 195 (50.39 %) females (Table 1). All the surveyed children 387 (100 %) reported to have been treated for intestinal schistosmiasis previously. Treatment was mostly done in schools, 369 (95.35 %), while majority 154 (39.79 %) of the children received treatment twice in a year (Table 1).

**Table 1:** Socio-demographic, environment and health characteristics of study participants

Demographic characteristics *n* = 387		Family information *n* = 387	
Characteristic	n (%)	Characteristic	n (%)
Age group (years)		Household head level of education	
8–9	26 (6.72 %)	Primary incomplete	51 (13.18 %)
10–11	155 (40.05 %)	Primary complete	111 (28.68 %)
12–13	156 (40.31 %	Secondary	138 (35.66 %)
>13	50 (12.92 %)	College and above	13 (3.36 %)
Class/Standard		Don’t know	74 (19.12 %)
4	105 (27.13 %)	Household head Occupation	
5	101 (26.10 %)	Farming	220 (56.85 %)
6	99 (25.58 %)	Business	97 (25.06 %)
7	82 (21.19 %)	Employed	66 (17.05 %)
Gender		Don’t know	4 (1.03 %)
Female	195 (50.39 %)	Possessions at home	
Male	192 (49.61 %)	Electricity	32 (8.27 %)
		Radio	338 (87.34 %)
		Television	175 (45. 34 %)
		Phone	319 (82.43 %)
Environmental characteristics *n* = 387		Health characteristics *n* = 387	
House wall type at home		Treated for worms	
Stone or bricks or cement	145 (37.47 %)	Yes	387 (100 %)
Clay or mud	113 (29.20 %)	No	0 (0 %)
Wood	109 (28.17 %)	Place of treatment	
Iron sheets	20 (5.17 %)	School	369 (95.35 %)
House floor type at home		Health Centre	11 (2.84 %)
Cement or tiles	88 (22.74 %)	Home	7 (1.81 %)
Wooden planks	2 (0.52 %)	Number of times treated	
Earth or sand	297 (76.74 %)	Once	97 (25.06 %)
Main water source at home		Twice	154 (39.79 %)
Piped/tap water	100 (25.84 %)	Thrice	94 (24.29 %)
Borehole or well	58 (14.99 %)	More than thrice	42 (10.85 %)
Rainwater	5 (1.29 %)	Ever been absent from school	
Stream or river	64 (16.54 %)	Yes	239 (61.76 %)
Canal	160 (41.34 %)	No	148 (38.24 %)
		Outcome when last visited health centre	
		Malaria	179 (46.25 %)
		Bilhazia	53 (13.70 %)
		Diarhorea	101 (26.10 %)
		Typhoid	17 (4.39 %)
		Others	37 (9.56 %)

### Prevalence of *S. mansoni*

The overall prevalence of *S. mansoni* was 53.7 %, (95%CI: 49.0–59.0). Prevalence was relatively high among all the surveyed schools with Mianya primary school showing the highest infection 75 % (95%CI: 67.3–83.6). While, Mukou and Mbui Njeru schools showed prevalence of 47.4 % (95%CI: 39.6–56.7) and 43.8 % (95%CI: 36.5–52.7) respectively. The male gender showed higher prevalence of the infection, 66.1 (95%CI: 59.8–73.2) compared to the female gender. More than half, 61.9 % (95%CI: 53.3–71.9) and 58.5 % (95%CI: 48.8–70.2) of the children in class 4 and class 7 respectively were infected with *S.mansoni*. There were relatively high infection levels among all the age groups with more than 50 % (Table 2).

**Table 2:** Prevalence of *S. mansoni*

Category	Prevalence (%)	95 % CI
Overall prevalence	53.7 %	49.0–59.0
Prevalence by school		
Mbui Njeru	43.8 %	36.5–52.7
Mianya	75.0 %	67.3–83.6
Mukou	47.4 %	39.6–56.7
Prevalence by location		
Thiba	45.5 %	40.0–51.8
Kangai	75.0 %	67.3–83.6
Prevalence by gender		
Female	41.5 %	35.2–49.1
Male	66.1 %	59.8–73.2
Prevalence by class		
4	61.9 %	53.3–71.9
5	46.5 %	37.8–57.4
6	48.5 %	39.6–59.4
7	58.5 %	48.8–70.2
Prevalence by age group		
8–9	53.8 %	37.7–76.9
10–11	51.0 %	43.7–59.5
12–13	55.8 %	48.5–64.1
>13	56.0 %	43.8–71.6

**Fig 2:**
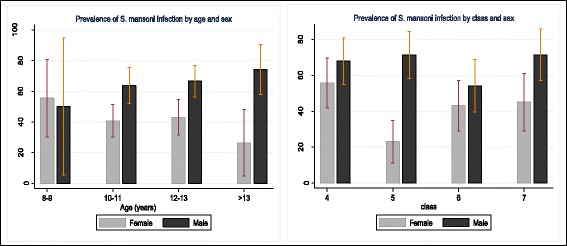
Prevalence of *S. mansoni* infection by age, sex and class

Further analysis of the data, revealed that prevalence of *S. mansoni* infection was influenced by age and class among both the boys and girls. In both gender, the prevalence of the infection varied significantly by age groups (*p* < 0.001). Those boys aged >13 years and girls aged (8–9) years, had the highest prevalence. The prevalence of *S.mansoni* in girls seemed to be decreasing with age while that of boys was increasing with age. Similarly, the prevalence varied significantly with the class of the child (*p* < 0.001), with prevalence of boys highest among class 7 pupils while prevalence of girls was highest among class 4 pupils (Fig. 2).

### Factors associated with *S. mansoni* infection

In this study, it was observed that male children were significantly more likely to be infected with *S. mansoni* compared to females (OR = 2.73, 95%CI: 2.03–3.65). Children who drew water from borehole, canal or stream/river were all significantly at risk of *S. mansoni* infection compared to those who use piped water (*p* < 0.001).In an attempt to determine the factors contributing to *S. mansoni* infection, the *forward stepwise variable selection method* was performed based on likelihood ratio (LR) test on the multivariable logistic regression inputting all the above factors in the model (see Table 3). The gender (sex) of a child was the only factor reported to be significantly associated with *S. mansoni* infection, (OR = 1.9, p-value = 0.015, 95%CI: 1.13–3.21).

**Table 3:** Fixed effects estimates for logistic regression model for *S. mansoni*

Multivariable logistic regression				
Factor		OR	p-value	95%CI
Gender	Male vs Female	2.73	<0.001*	2.03–3.65
Class	4 vs 7	1.30	0.347	0.75–2.24
	5 vs 7	0.67	0.089	0.42–1.07
	6 vs 7	0.68	0.063	0.45–1.02
Age Category	(8–9) vs >13	0.90	0.798	0.41–1.98
	(10–11) vs >13	0.92	0.765	0.55–1.55
	(12–13) vs >13	1.09	0.681	0.72–1.65
House wall type	Mud vs Stone/bricks	1.66	0.003*	1.19–2.31
	Wood vs Stone/bricks	1.43	0.038*	1.02–2.00
	Iron sheets vs Stone/bricks	0.62	0.143	0.32–1.17
House floor type	Earth vs Cement	0.82	0.288	0.56–1.19
Water source at home	Borehole/well vs Piped water	2.20	0.001*	1.40–3.46
	Canal vs Piped water	2.61	<0.001*	1.87–3.65
	Rainwater vs Piped water	2.25	0.261	0.54–9.27
	Stream/River vs Piped water	2.93	<0.001*	1.93–4.44
Round of treatment	1 vs >3	0.48	0.047*	0.24–0.99
	2 vs >3	0.55	0.064	0.29–1.04
	3 vs >3	0.65	0.224	0.32–1.31
Disease diagnosed with when ill last term	Bilharzia vs Others	2.38	0.068	0.94–6.06
	Diarrhea vs Others	1.12	0.814	0.44–2.84
	Malaria vs Others	0.74	0.260	0.44–1.25
	Stomach ache vs Others	0.94	0.820	0.56–1.59
	Typhoid vs Others	1.28	0.698	0.36–4.51
Time to nearest health centre	60 min vs <30 min	0.87	0.482	0.60–1.27
	>60 min vs <30 min	1.18	0.657	0.56–2.50
Disease when last visited hospital	Bilharzia vs Others	1.11	0.808	0.49–2.53
	Diarrhea vs Others	0.82	0.593	0.40–1.69
	Malaria vs Others	0.83	0.596	0.42–1.64
	Typhoid vs Others	1.30	0.628	0.44–3.82

## Discussion

The present study demonstrated that there was continued intestinal schistosomiasis infection in the school children during the MDA programme and two years following its withdrawal. It is evident that during the programme, much emphasis was put on treatment, neglecting other essential components of *S. mansoni* control like provision of clean water, sanitary facilities, health education and personal hygiene. Other attributing factors could be non treatment of community members (adults) who are potentially infected and lack of control of the intermediate hosts (snails). Indeed, in a study conducted in coastal part of Kenya, showed that most control programmes/activities of schistosomiasis often target school-age children only, leaving out the rest of the community who are equally infected and may act as a reservoir for transmission and a source of re-infections to the school-age children [35]. Accordingly, we recommend that future control programmes should not only focus on chemotherapy but also on other components of *S. mansoni* control.

It was observed that the gender (sex) of a child was a risk factor significantly influencing *S. mansoni* infection in the study subjects, (OR = 1.9, p-value = 0.015, 95%CI: 1.13–3.21). This could probably be due to boys are more adventurous than girls and they may come into contact with freshwater bodies infested with cercariae that have been released by intermediate host snails while playing or swimming unlike girls who are culturally restricted. This corroborates with a study conducted in Mbita, western Kenya, which reported that male children were highly infected with *S.mansoni* compared to female children due to their play habits [36].

Further analysis showed that children who drew water from canal, borehole or stream/river were significantly at risk of *S. mansoni* infection compared to those who use piped water (*p* < 0.001). These variations might have been due to differences in environmental sanitation and educational status of parents’ study subjects. We also suggest that this could be attributed to frequent visit to the water bodies which might be infested with *S. mansoni* parasites, hence more exposure to infection. These findings agree with previous research which clearly indicated that environmental living circumstances were tightly connected with infection status and disease burden [37]. This calls for additional ecological and environmental survey to understand the distribution and population dynamics of snail intermediate host which directly relates with the transmission of schistosomes.

The major potential weakness of this study may be the fact that only one stool sample was collected. The accuracy of the Kato-Katz technique in identifying individuals with *S. mansoni* infections is limited by day-to-day variation in egg excretion and sensitivity is greatly reduced when intensity of infections is low [13]. Improved detection of *S. mansoni* eggs in stool requires examination of stool specimens collected on 2 to 3 consecutive days which may not be practical especially when working in very remote areas. A new technique known as FLOTAC has been proposed as a better tool for diagnosis of parasitic infections like *S. mansoni*. A study conducted in Cote d’Ivoire, the FLOTAC technique was found to have a sensitivity of 88.2 % compared with 68.4 % for Kato-Katz [14].

## Conclusions

The analysis of collected data provided insight into the current prevalence status of *S. mansoni* infections and the associated risk factors two years after withdrawal of MDA programme. The results of this study suggest that it may be necessary to develop approaches which offer continued preventive chemotherapy interventions in high endemic areas to reduce new infections or re-infections. We also recommend that future control programmes should consider including adult community members, accompanied by other essential aspects of *S. mansoni* control.

## Abbreviations

JICA: Japan international cooperation agency
KEMRI: Kenya medical research institute
STHs: Soil transmitted helminthes
WHA: World health assembly
ESACIPAC: Eastern and Southern Africa centre of international parasite control
WHO: World Health Organization
MDA: Mass drug administration
*S.mansoni*: *Schistosoma mansoni*
EPG: Eggs per gram
PPS: Probability proportional to size
MOH: Ministry of health
OR: Odds ratio
